# The tumor microenvironment of non–small cell lung cancer impairs immune cell function in people with HIV

**DOI:** 10.1172/JCI177310

**Published:** 2025-06-03

**Authors:** Shruti S. Desai, Syim Salahuddin, Ramsey Yusuf, Kishu Ranjan, Jianlei Gu, Lais Osmani, Ya-Wei Lin, Sameet Mehta, Ronan Talmon, Insoo Kang, Yuval Kluger, Hongyu Zhao, Kurt Schalper, Brinda Emu

**Affiliations:** 1 Department of Pathology, Yale University School of Medicine, New Haven, Connecticut, USA.; 2 Alexion Pharmaceuticals, Boston, Massachusetts, USA.; 3 Department of Internal Medicine, Section of Infectious Diseases, Yale University School of Medicine, New Haven, Connecticut, USA.; 4 University of Iowa Carver College of Medicine, Iowa City, Iowa, USA.; 5 Yale University School of Medicine, New Haven, Connecticut, USA.; 6 Department of Ophthalmology, University of Miami Jackson Health System, Miami, Florida, USA.; 7 Department of Biostatistics and; 8 Department of Internal Medicine, Section of Rheumatology, Yale University School of Medicine, New Haven, Connecticut, USA.; 9 Department of Electrical and Computer Engineering Technion-Israel Institute of Technology, Haifa, Israel.; 10 Yale Center for Genome Analysis, Yale University School of Medicine, New Haven, Connecticut, USA.

**Keywords:** AIDS/HIV, Immunology, Oncology, Lung cancer

## Abstract

Lung cancer is the leading cause of cancer mortality among people with HIV (PWH), with increased incidence and poor outcomes. This study explored whether the tumor microenvironment (TME) of HIV-associated non–small cell lung cancer (NSCLC) limits tumor-specific immune responses. With a matched cohort of NSCLC samples from PWH and from people without HIV (PWOH), we used imaging mass cytometry, a linear mixed-effects model, and an artificial intelligence–based (AI-based) PageRank mathematical algorithm based on spectral graph theory to demonstrate that HIV-associated tumors have differential distribution of tumor-infiltrating CD8^+^ and CD4^+^ T cells, enriched for the expression of programmed cell death 1 (PD-1) and lymphocyte-activating gene 3 (LAG3), as well as activation and proliferation markers. We also demonstrate higher expression of immunoregulatory molecules (PD-L1, PD-L2, B7-H3, B7-H4, IDO1, and VISTA) among tumor-associated macrophages. Discrimination of cells between tumors from PWH versus those from PWOH was confirmed by spectral graph theory with 84.6% accuracy. Furthermore, we noted differences in spatial orientation of immune cells within the TME of PWH compared with PWOH. Additionally, cells from PWH, compared with those from PWOH, exhibited decreased tumor killing when exposed to HLA-matched NSCLC cell lines. In conclusion, our study demonstrates that the HIV-associated TME sustained a unique immune landscape, showing evidence of immune cells with enhanced immunoregulatory phenotypes and impaired antitumor responses, with implications for responses to immune checkpoint blocker therapies.

## Abstract 2

This study determined that the tumor microenvironment of lung cancers from people with HIV reveals a more immunoregulatory environment compared to people without HIV.

## Introduction

Non-AIDS-defining cancers (NADCs) have overtaken AIDS-defining malignancies as the leading cause of cancer-related deaths among people with HIV (PWH) in the United States. Lung cancer has emerged as the most common NADC and a leading cause of overall mortality among PWH (1). As many as 40% of all cancer deaths occur as a result of lung cancer, as well as 5% of all deaths for PWH (2, 3). The overall risk of lung cancer has consistently been demonstrated to be greater in PWH than in people without HIV (PWOH) infection, with as high as a 3- to 4-fold increased risk, an effect independent of active combined antiretroviral treatment (cART), serum CD4^+^ T cell counts, or CD4^+^ T cell nadir values (4–6). As in PWOH, the vast majority of lung malignancies in PWH comprise the non–small cell lung cancer (NSCLC) subsets adenocarcinoma and squamous cell carcinoma, but they occur at a younger age and present with a more advanced clinical stage than in the general population (1, 4, 7).

HIV infection results in depletion of CD4^+^ T cells, predisposing PWH to opportunistic infections and AIDS-defining cancers. However, CD4^+^ T cell depletion is not the sole immune perturbation seen in chronic HIV infection. Immune activation and global immune dysfunction are also hallmarks of HIV infection, phenomena only partially reversible with initiation and maintenance of cART (8). It is currently unknown how global immune dysregulation in the setting of HIV infection affects local antitumor immune responses and/or tumor cell properties, and how this may affect prognosis and treatment-specific outcomes in patients with lung cancer.

The tumor immune microenvironment in NSCLC is a spatially complex array of multiple cell types including malignant epithelial cancer cells, nonmalignant stromal cells, and diverse immune cell subsets, with implications for prognosis and response to treatment (9–12). The prominent role of local T cell responses in tumor elimination is highlighted by the clinical success of therapies blocking key immune-inhibitory receptors upregulated in antigen-activated T cells such as CTLA-4 and the programmed cell death 1/programmed death ligand 1 (PD-1/PD-L1) pathways (collectively referred to as immune checkpoint blockers [ICBs]). However, most patients with lung cancer are immunocompetent, and tumors are expected to develop in the presence of adaptive immune pressure. In PWH, however, the normal interactions between a neoantigen and evolution of an effective immune response may be impaired.

Multiple studies have shown that the tumor microenvironment (TME) composition is a key determinant of cancer survival and sensitivity to ICBs. For instance, the level of local PD-L1 expression, the density of tumor-infiltrating lymphocytes (TILs), and the functional profile of TILs are associated with clinical benefit to PD-1 axis blockers in patients with advanced NSCLC (9–12). Using single-cell and spatially resolved analysis of human NSCLCs, we have previously characterized local effector T cell dysfunction and found that evidence of enhanced T cell proliferation, terminal differentiation, and upregulation of immune-inhibitory receptors were associated with reduced sensitivity to PD-1 axis blockers among patients with NSCLC (10–12). Similar findings have been reported by others using single-cell RNA-Seq strategies (13). Recent studies have also revealed a prominent role of TIL spatial location in the tumor bed and emphasized the relevance of spatial immune heterogeneity in sensitivity/resistance to PD-1 axis blockers in NSCLC (14). In addition, an asymmetric distribution of TILs with the presence of cytotoxic T cells in the periphery of tumors (e.g., “leading edge”) with a relative absence in the tumor center characterizes poorly immunogenic malignancies, which has been associated with unfavorable outcomes (15–17). Collectively, these studies support a prominent role of TILs and the TME in NSCLC rejection of and response to immunotherapy.

The expected effect of HIV on T cells within the TME of NSCLC remains unknown. Several aspects of local immune dysfunction in NSCLC resemble those seen systemically in PWH, suggesting potentially additive or even synergistic defective consequences. In this comprehensive study of the immunologic components of the TME among PWH and NSCLC, we conducted a comparative analysis of HIV-associated and clinically matched NSCLC tumor samples from PWOH utilizing spatially resolved and multiplexed tissue imaging modalities. Here, we show that the TME of HIV-associated NSCLC had several features of immune exhaustion, increased T cell proliferation, and increased tumor mutational burden, suggesting greater dysfunction in the context of HIV-associated NSCLC compared with HIV infection or NSCLC alone. We additionally show that circulating T cells from PWH, compared with T cells from HLA-matched PWOH, had poor tumor killing and enhanced upregulation of T cell–inhibitory receptors when exposed to lung tumor cell lines.

## Results

### PWH and PWOH were matched for clinicopathologic characteristics.

NSCLC tumor samples from 18 PWH and 19 PWOH were clinicopathologically matched and included in the analysis. Histologic subtype, stage at cancer diagnosis, year of cancer diagnosis, age, sex, and smoking status were comparable between the 2 groups (*P >* 0.05; Supplemental Tables 2 and 3; supplemental material available online with this article; https://doi.org/10.1172/JCI177310DS1). The median age among PWH and PWOH was 54 and 57 years, respectively. For PWH, the median CD4^+^ T cell count was 440 cells/μL, 77% were on cART at the time of their cancer diagnosis, and 44% had an undetectable viral load (<400 copies/μL). The median time since HIV diagnosis was 17 years, and 56% of the PWH had an AIDS diagnosis. Of note, PWH had significantly decreased overall survival compared with PWOH (median survival of 351 vs. 2,451 days, *P* = 0.037, Supplemental Figure 1A). PWH with a suppressed viral load also had decreased overall survival compared with PWH with a detectable HIV load (>400 copies/mcL) at the time of the cancer diagnosis (*P* = 0.20, Supplemental Figure 1B), but the cause of mortality was unknown for these individuals.

### Lymphocytes and macrophages infiltrate the TME of individuals with HIV-associated NSCLC.

**Figure 1:**
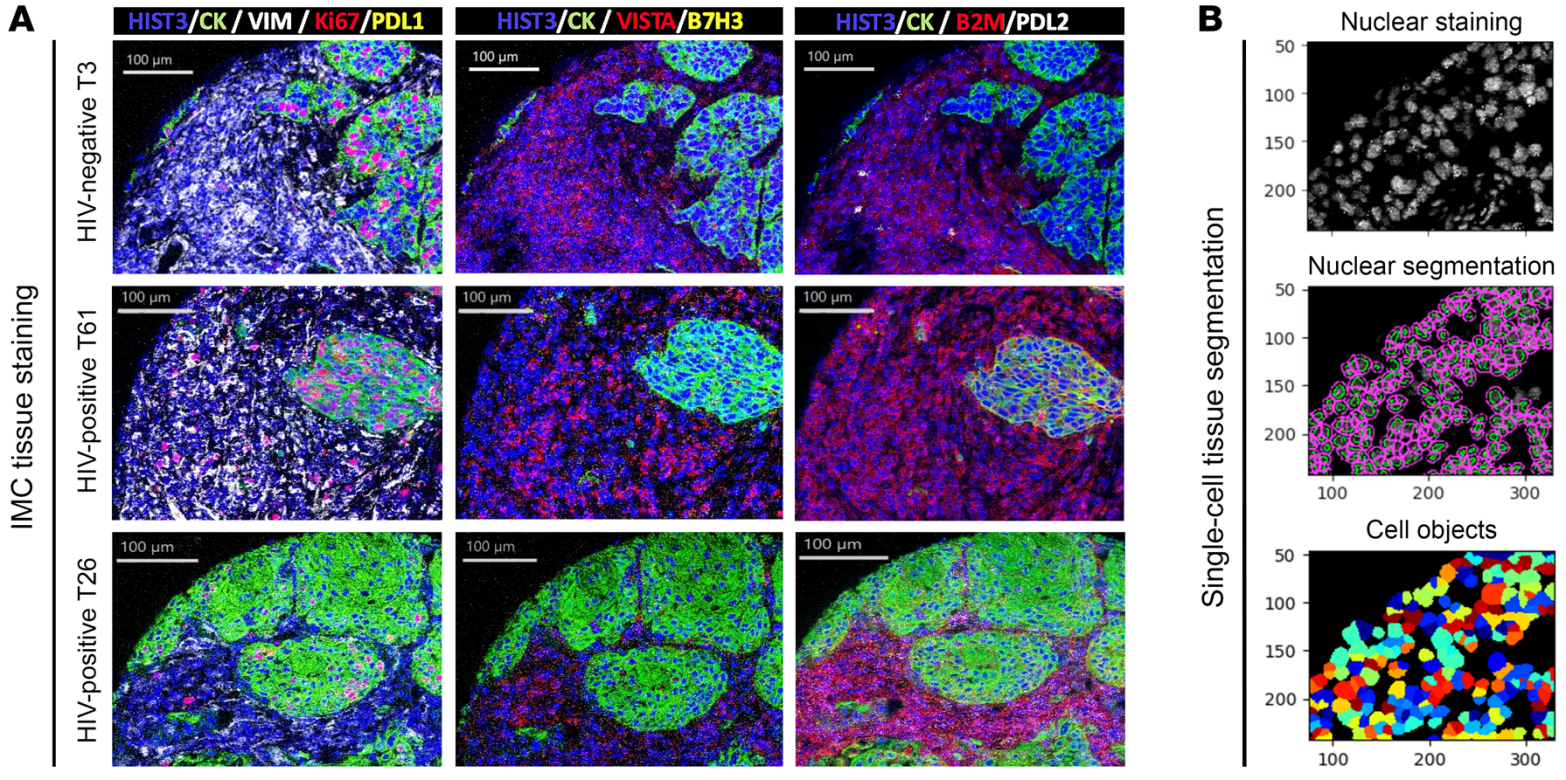
IMC staining and segmentation. (**A**) Representative multicolor microphotographs of a single NSCLC tumor stained with a 37-marker IMC panel. Captions of the same tumor display different markers, as indicated by the color code for each panel. Scale bars: 100 μm. (**B**) Representative image showing the single-cell segmentation of IMC tumor images using nuclear cell markers and digital cell objects. Original magnification, ×10.

We studied the NSCLC samples using a standardized imaging mass cytometry (IMC) panel for simultaneous visualization and spatially resolved measurement of 37 protein markers. These markers included cell phenotype indicators (e.g., cytokeratin [CK], CD3, CD4, CD8, CD68, CD-19), functional markers and potential candidate immunotherapy targets (e.g., GZB, PD-1, LAG3, TIM3, KI67, PD-L1, PD-L2, VISTA, IDO-1), and structural indicators for the tissue compartment (vimentin) and cell segmentation (histone 3 and DNA intercalators). Representative images from the IMC panel staining are shown in Figure 1A. As expected, CD3^+^ TILs and CD68^+^ tumor-associated macrophages (TAMs) were predominantly located in the CK^–^ stromal tissue areas and showed variable marker profiles. Tumor cells showed prominent CK positivity and focal expression of immunomodulatory markers such as β2-microglobulin, PD-L1, and PD-L2. After staining and acquisition, the tumor images from all samples were digitally segmented into single-cell populations using nuclear segmentation models and expression of phenotypic cell markers (Figure 1B).

**Figure 2:**
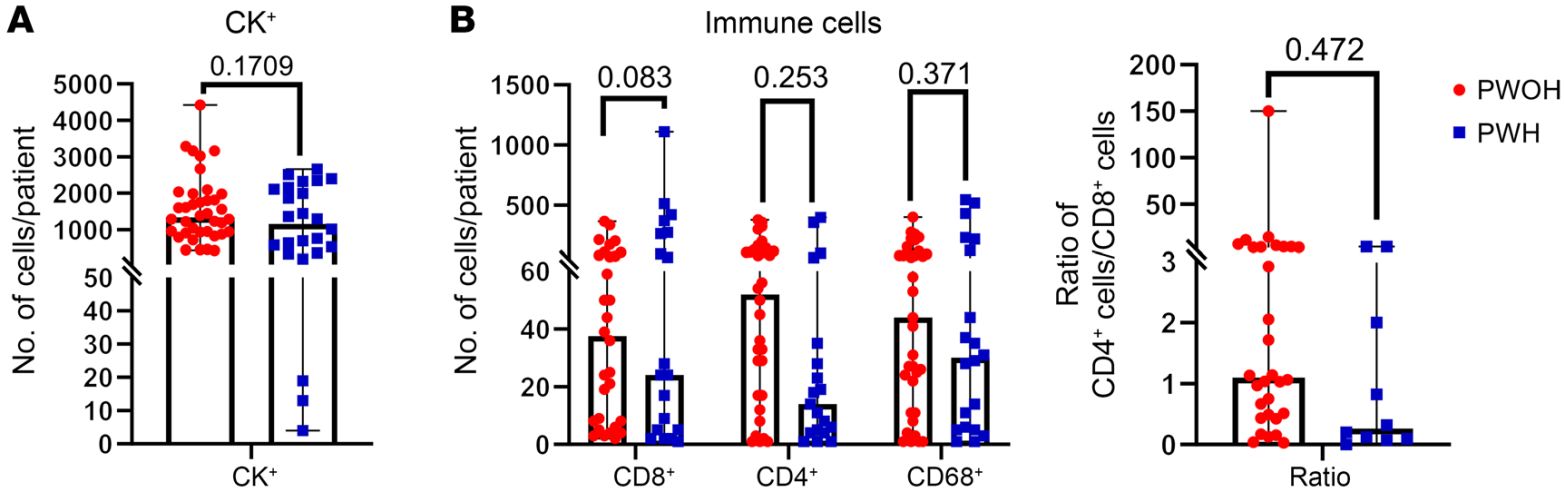
Immune cell infiltration into the TME. (**A**) Mean number of CK^+^ tumor cells, CD8^+^ and CD4^+^ TILs, and CD68^+^ TAMs in non-HIV controls (red bars) and in HIV-associated NSCLCs (blue bars). Each bar depicts the mean ± SEM, and each colored symbol represents an individual patient. (**B**) CD4^+^/CD8^+^ TIL ratio in control non-HIV NSCLC patient samples (red bar) and HIV-associated NSCLC patient samples (blue bar). Each bar depicts the mean ± SEM.

Quantification of main cell subpopulations of more than 35,000 individual cells obtained from patients/controls in the cohort showed comparable levels of CK^+^ tumor cells and CD68^+^ TAMs (Figure 2, A and B) between PWH and PWOH. There was a trend toward lower CD4^+^ TIL numbers (*P* = 0.25), increased CD8^+^ TIL numbers (*P* = 0.08), and a decreased CD4^+^/CD8^+^ T cell ratio (*P* = 0.47) among HIV-associated NSCLCs, but the difference did not reach statistical significance.

The IMC findings were confirmed with quantitative immunofluorescence (QIF) staining and analysis, which also showed no overall differences between CD4^+^ and CD8^+^ T cells or B cell infiltration into the tumor environment (data not shown). Of note, compared with tumors from PWOH, PWH with uncontrolled HIV replication (viral load >400 copies/μL) had decreased numbers of TILs for CD4^+^ (*P* = 0.02) and CD20^+^ (*P* = 0.02) populations. Among HIV-associated NSCLC, age, cancer stage, AIDS status, and peripheral blood CD4^+^ T cell count were not associated with T cell infiltration in the TME (data not shown).

### TILs display altered functional profiles in HIV-associated NSCLC.

IMC single-cell data were analyzed to identify and compare salient features of CD8^+^ and CD4^+^ TIL subpopulations in tumors from PWH and PWOH. The markers included differentiation (TBET, CD45RO), activation (CD25, PD-1, and granzyme B [GRZB], proliferation (KI67), and immune-inhibitory receptors associated with T cell exhaustion (PD-1, LAG3, and T cell immunoglobulin and mucin domain-containing protein 3).

**Figure 3:**
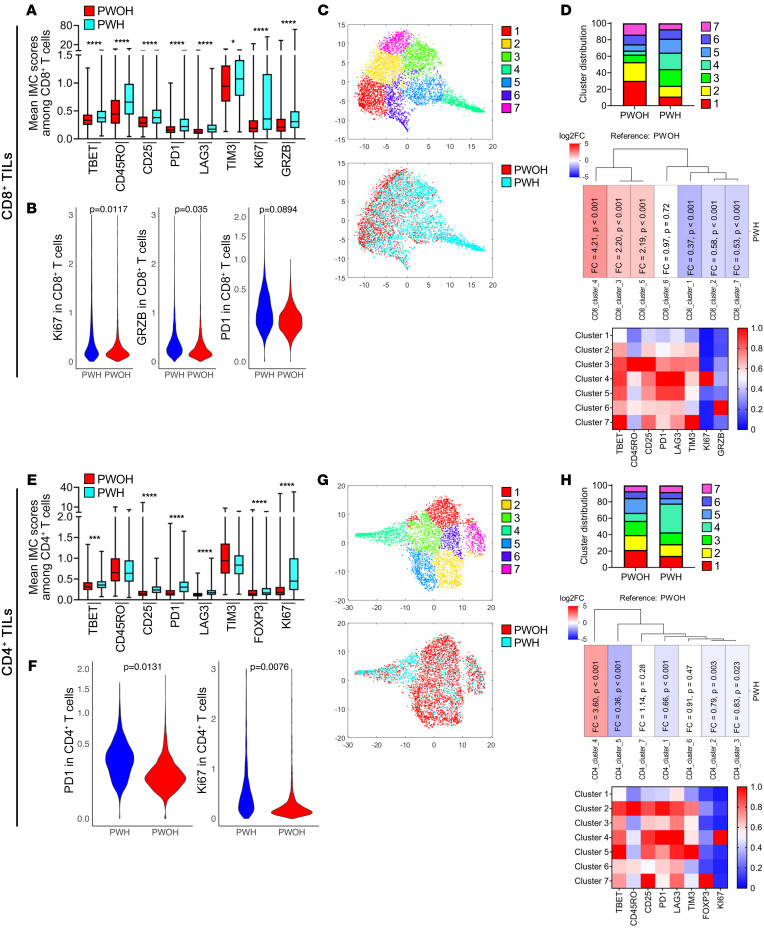
T cell phenotype in the TME of NSCLC in PWH and PWOH. (**A** and **E**) Mean expression value of single markers in CD8^+^ T cells (**A**) and CD4^+^ T cells (**E**) from IMC panels. *P < 0.05, *** *P* < 0.001, and ****P < 0.0001, by nonparametric Kruskal-Wallis test. Data are shown as the mean ± SEM. (**B** and **F**) Umbrella plots revealed markers with significant differences between PWH and PWOH with regard to CD8^+^ T cells (**B**) and CD4^+^ T cells (**F**), using a random-effects model. (**C** and **G**) Unsupervised clustering analysis reveals 7 unique clusters of CD8^+^ (**C**) and CD4^+^ (**G**) T cells as a *t*-distributed stochastic neighbor embedding (*t*-SNE) plot (top panels) and the distribution of cells by HIV status with cells from PWH (blue) and PWOH (red) (bottom panels). (**D** and **H**) Bar graphs representing the distribution of each CD8^+^ (**D**) and CD4^+^ (**H**) T cell cluster in tumors from PWH and PWOH. Fold-change graphs demonstrate the change in proportions for each subset in HIV-associated tumors from PWH compared with non-HIV tumors from PWOH. Heatmaps show the intensity of each marker across the different clusters, normalized from 0 to 1, with 1 being highest expression.

As shown in Figure 3A and after adjusting for multiple comparisons, CD8^+^ T cells from HIV-associated tumors showed significantly higher levels of CD45RO and TBET expression; increased KI67 expression; increased expression of activation markers CD25 and GRZB; as well as higher levels of the T cell exhaustion markers PD-1, LAG3, and TIM3. A random effects model, which additionally controlled for inter-patient variation, further confirmed significantly elevated levels of KI67 (1.62 vs. 0.53, *P* = 0.01) and GRZB (0.55 vs. 0.42, *P* = 0.04) in CD8^+^ TILs from PWH compared with those from PWOH, respectively, and a trend toward increased PD-1 expression (0.27 vs. 0.18, *P* = 0.09) (Figure 3B). Within the random effects model, there was no statistical difference in the levels of CD45R0, TBET, CD25, LAG3, or TIM3.

Beyond individual marker expression levels, which do not provide a holistic evaluation of T cell populations, we next asked whether phenotypic multimarker patterns of CD8^+^ TILs were different between tumors from PWH versus those from PWOH, using an unsupervised clustering algorithm. Seven unique CD8^+^ T cell clusters were generated on the basis of the patterns of marker expression that demonstrated a differential distribution between samples from PWH and PWOH (Figure 3C). Evaluation of the distribution of cells between the 2 groups revealed prominent differences, notably with expansion of clusters 3, 4, and 5. Cells in these 3 clusters represented 57.1% of CD8^+^ TILs among HIV-associated tumors, but only 21.7% in tumors from PWOH (*P* < 0.001). These 3 clusters were unique by definition, but they each had high relative expression of PD-1 and LAG3 comparatively, as well as lower levels of GRZB among all clusters. Cluster 3 was notable for its very high levels of CD45RO and CD25, suggestive of an “activated effector memory cell subset.” Cluster 4 strongly resembled previously described dysfunctional “effector burned-out cells” (Ebo cells) (12) which have high levels of PD-1 and LAG3, with elevated KI67 and low cytotoxic (GRZB) activity. An increase in the presence and expansion of these dysfunctional Ebo cells has recently been associated with limited clinical benefit from PD-1 axis blockers in patients with advanced NSCLC. Here, we show that these Ebo cells were even further expanded in tumors from PWH compared with tumors from PWOH (19.9% of all CD8^+^ T cells among HIV-associated tumors, whereas this cluster comprised only 4.7% of CD8^+^ TILs among uninfected controls, *P* < 0.001). Cluster 5, which also appeared “highly exhausted but nonproliferating” with elevated expression of PD-1 and LAG3 closely resembled Ebo cells but without elevated KI67 expression. Of note, among tumors from PWOH, the majority of CD8^+^ TILs were found within clusters 1 and 2 (52.9% of the total) compared with only 24.3% among the HIV- associated tumors. Clusters 1 and 2, which were decreased among PWH compared with PWOH (*P* < 0.001), were notable for their relatively low expression of PD-1 and LAG3, as well as their higher expression of CD45RO, suggesting that these cells were less differentiated and representing a more functional central memory phenotype. Despite the small patient numbers, when we controlled for individual patient variation in the distribution of clusters, clusters 4 and 5 confirmed the trend toward increased proportions in PWH compared with PWOH, and clusters 1 and 2 continued to show decreased proportions in PWH, with a significant difference noted in cluster 1 (*P* = 0.05, Supplemental Figure 2A).

Single-cell expression levels of the phenotypic and functional markers analyzed among CD4^+^ TILs (Figure 3E) showed patterns similar to those seen among CD8^+^ TILs, with higher levels of the activation markers CD25 and TBET, the T cell–inhibitory receptors PD-1 and LAG3, as well as the proliferation marker KI67 in tumors from PWH compared with those from PWOH. Notably, unlike CD8^+^ TILs, we observed no difference in CD45RO or TIM3 expression between the 2 groups. In a random-effects model, PD-1 (0.34 vs. 0.19, *P* = 0.01) and KI67 (1.60 vs.. 0.56, *P* = 0.008) expression remained significantly elevated among CD4^+^ T cells in tumors from PWH compared with those from PWOH (Figure 3F).

Unsupervised clustering revealed 7 unique clusters which, like in the CD8^+^ T cell population, showed differential distribution between HIV-associated and non-HIV tumors (Figure 3, G and H). Most striking is the finding that cluster 4 was most predominant among CD4^+^ T cells from HIV-associated tumors, making up 35.2% of all CD4^+^ TILs in the TME, whereas, among non-HIV tumors, cluster 4 only comprised 9.8%. Detailed characterization of cluster 4 again revealed very high expression of PD-1, LAG3, as well as KI67, suggesting a dysfunctional profile, as recently reported (18–20). Thus, this phenotype mirrored the Ebo phenotype seen in CD8^+^ T cells and further exhibited elevated CD25 expression, indicating an activated state. Cluster 7 likely represented the Treg population, given the high expression levels of FoxP3 and CD25, and was found in comparable proportions in both HIV-associated and non-HIV tumors, at 8.0% and 7.0%, respectively, despite our observation that expression of the single marker FoxP3 was slightly higher in HIV-associated CD4^+^ T cells. Controlling for inter-patient variation, we found that the CD4^+^ cluster 4 remained increased in tumors from PWH compared with those from PWOH (Supplemental Figure 2B). Additionally, there was no significant difference in the distribution of clusters among patients with or without control of HIV replication (HIV load ≥400 copies/μL vs. >400 copies/μL; data not shown).

### TAMs show distinct immunoregulatory features in HIV-associated tumors.

**Figure 4:**
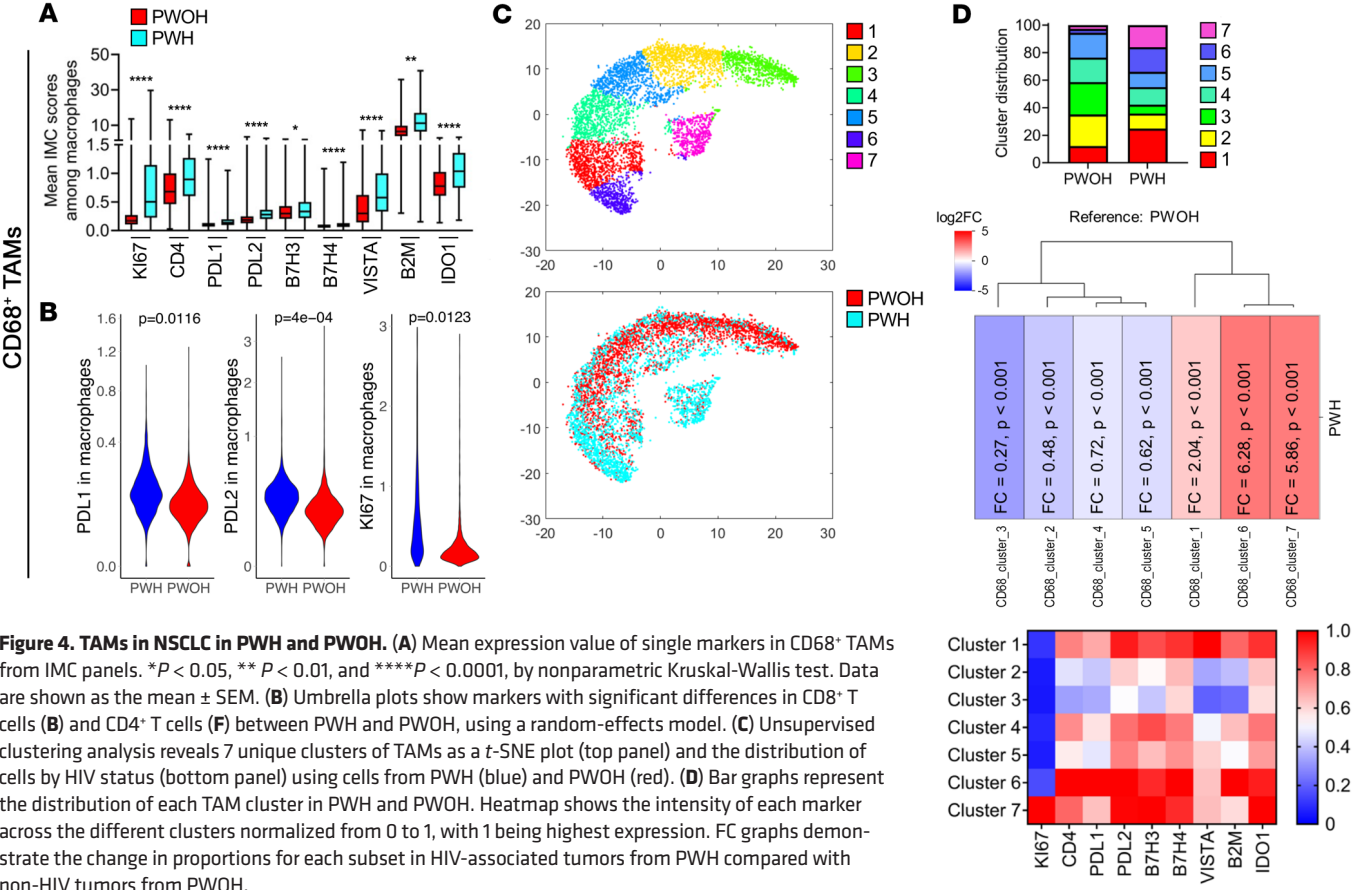
TAMs in NSCLC in PWH and PWOH. (**A**) Mean expression value of single markers in CD68^+^ TAMs from IMC panels. *P < 0.05, ** *P* < 0.01, and ****P < 0.0001, by nonparametric Kruskal-Wallis test. Data are shown as the mean ± SEM. (**B**) Umbrella plots show markers with significant differences in CD8^+^ T cells (**B**) and CD4^+^ T cells (**F**) between PWH and PWOH, using a random-effects model. (**C**) Unsupervised clustering analysis reveals 7 unique clusters of TAMs as a *t*-SNE plot (top panel) and the distribution of cells by HIV status (bottom panel) using cells from PWH (blue) and PWOH (red). (**D**) Bar graphs represent the distribution of each TAM cluster in PWH and PWOH. Heatmap shows the intensity of each marker across the different clusters normalized from 0 to 1, with 1 being highest expression. FC graphs demonstrate the change in proportions for each subset in HIV-associated tumors from PWH compared with non-HIV tumors from PWOH.

TAMs are recognized as increasingly important in the orchestration of antitumor immunity in the TME. Evaluation of CD68^+^ TAMs revealed differential results between HIV-associated and non-HIV tumors. Evaluation of individual markers showed that HIV-associated tumors had increased levels of the immunoregulatory receptors PD-L1, PD-L2, B7H3, B7H4, and VISTA (Figure 4A). The differences between cell-surface expression were confirmed for PD-L1 (0.16 vs. 0.11, *P* = 0.01), PD-L2 (0.30 vs. 0.20, *P* < 0.0001), and KI67 (1.43 vs. 0.36, *P* = 0.01) among TAMS from PWH versus PWOH, respectively, using a random-effects model (Figure 4B). In addition, TAMs in HIV-associated tumors have evidence of increased IDO1, an enzyme that converts tryptophan to kynurenine, a potent immunosuppressive molecule. And, as with T cells, TAMs in HIV-associated tumors had significantly increased KI67 expression. These findings point to HIV-associated NSCLCs harboring TAMs that are associated with universal upregulation of markers associated with an immunoregulatory phenotype, known to render T cells less functional. Of note, β-2 microglobulin, a marker of immune activation and antigen presentation, was also elevated in TAMs from HIV-associated tumors. Unsupervised clustering of TAMs by cumulative expression of multiple markers also demonstrated that TAMs from HIV-associated versus non-HIV tumors were distinct (Figure 4C), with clusters 1, 6, and 7 making up 58.8% of TAMs from HIV-associated tumors but only 17.8% of TAMs from non-HIV tumors. Clusters 6 and 7 were notable for their coexpression of multiple markers from the B7 family including PD-L1, PD-L2, B7H3, and B7H4, whereas cluster 1 was notable for its high expression of VISTA. This was in stark contrast to TAMs from non-HIV tumors, in which clusters 2, 3, 4, and 5 made up 82.2% of the TAM population (Figure 4D), and these clusters were notable for their lower expression of each of the immunoregulatory molecules identified. In an analysis that evaluated the proportions of these clusters among individual patients, the trend toward increased proportions of cluster 1 in PWH was evident, as well as decreased proportions of clusters 2, 3, and 5, with a significant difference noted in cluster 3 (*P* = 0.05, Supplemental Figure 2C).

### Spectral graph theory confirms the ability to discriminate the TME of HIV-associated NSCLC from that of non-HIV NSCLC.

**Figure 5:**
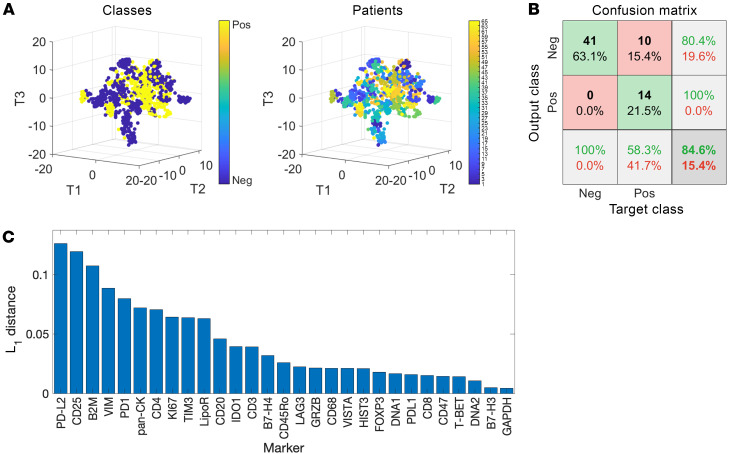
Spectral graph theory to distinguish HIV from non-HIV NSCLC TME. (**A**) Visualization of patches representation in 3D using *t*-SNE, colored according to HIV status (Classes) and patients. (**B**) Confusion matrix for HIV prediction obtained using a leave-one-subject-out cross-validation based on a RBF SVM classifier. (**C**) Antibodies ranked according to the L1 distance between their SSDs on the graph of 14 patches associated with the highest CK expression levels. Pos, positive; Neg, negative.

Cell segmentation analysis algorithms are critically important for the identification of potential novel cell subsets within complex datasets such as IMC. However, in order to confirm our artificial intelligence–based (AI-based) cell segmentation and quantitative platforms, we additionally used an alternative approach to imaging analysis. A computational strategy based on the PageRank mathematical algorithm was used in order to establish an unsupervised and cell segmentation–independent signature associated with HIV status (21). First, 14 individual 35 mm^2^ micro-image patches were selected per patient using the highest expression of lineage-defining cell markers (CD4, CD8, and CK cells). Within each patch, tumor and neighboring immune cells were mapped with consideration of all markers. For each patch, steady-state distribution (SSD) vectors were computed, organized into a covariance matrix, subjected to nonlinear dimensionality reduction using diffusion maps, and classified using a radial basis function (RBF) support vector machine (SVM) classifier. Finally, a leave-one-case-out cross-validation analysis was performed to show accuracy for associations with a group. The integrated spatial classifier was applied to the cohort of HIV and non-HIV cases. Figure 5A demonstrates the clustering of patients, in which each point identifies a single patch, color-coded by HIV status. The corresponding confusion matrix was attained by a classifier applied to the low-dimensional representation obtained by diffusion maps. By training and testing the classifier as described above, HIV-associated NSCLC could be distinguished from non-HIV NSCLC, providing discrimination with 84.6% accuracy (Figure 5B). The proteins most likely to differentiate HIV-associated from non-HIV in rank order were PD-L2, CD25, β2M, vimentin, and PD-1 (Figure 5C). The majority of markers associated with differentiating HIV-associated versus non-HIV tumors using spectral graph theory were the same markers that differentiated individual immune subsets (PD-L2 expression in TAMS, PD-1 and CD25 expression in T cells) using the cell segmentation approach to IMC analysis. This concordance, utilizing 2 different algorithms and approaches to measure complex IMC data, provides confidence that the expression levels of these specific markers do in fact discriminate the TME of HIV-associated versus non-HIV NSCLC. In addition, some markers found on CK^+^ tumor cells, including β2-microglobulin and vimentin, aided in the discrimination of HIV versus non-HIV-associated NSCLC cells, revealing future areas to explore that include differences in protein expression of tumor cells in PWH compared with those of PWOH.

### Spatial architecture reveals increased distances between immune cells in the HIV-associated TME.

**Figure 6:**
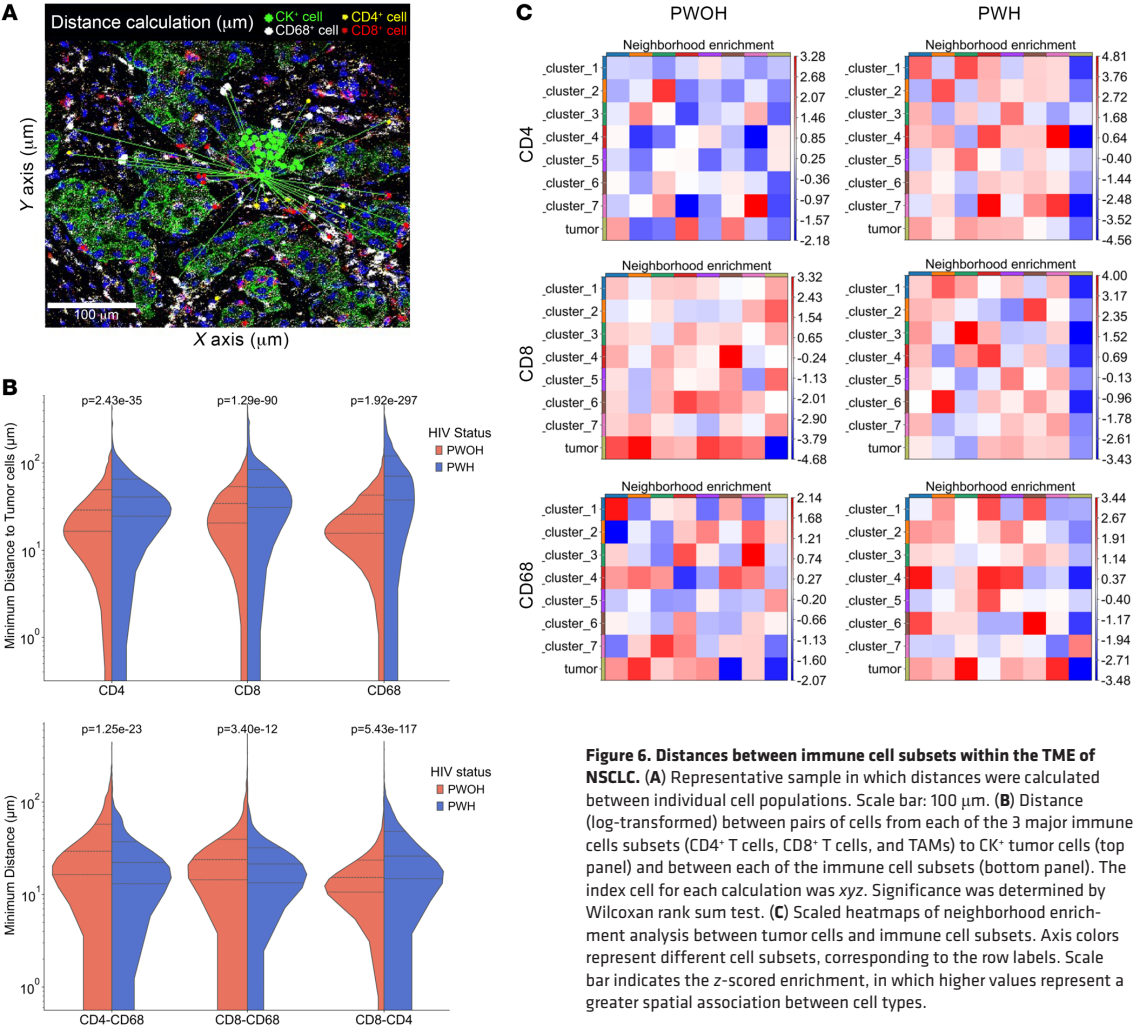
Distances between immune cell subsets within the TME of NSCLC. (**A**) Representative sample in which distances were calculated between individual cell populations. Scale bar: 100 μm. (**B**) Distance (log-transformed) between pairs of cells from each of the 3 major immune cells subsets (CD4^+^ T cells, CD8^+^ T cells, and TAMs) to CK^+^ tumor cells (top panel) and between each of the immune cell subsets (bottom panel). The index cell for each calculation was *xyz*. Significance was determined by Wilcoxan rank sum test. (**C**) Scaled heatmaps of neighborhood enrichment analysis between tumor cells and immune cell subsets. Axis colors represent different cell subsets, corresponding to the row labels. Scale bar indicates the *z*-scored enrichment, in which higher values represent a greater spatial association between cell types.

An attractive feature of IMC, in additional to the ability to use a large number of phenotypic and functional markers for cell characterization, is the ability to maintain spatial architecture. The distribution of immune cells within and surrounding tumor cells is of increasing interest and has been noted to be associated with prognosis in some studies. Using our IMC data, which preserved the spatial architecture of the TME, we calculated the distances between individual immune cell populations (CD4^+^ and CD8^+^ TILs, and CD68^+^ TAMs), as well as the distances between those immune cell populations and tumor cells (Figure 6A). Using a method that measured the minimum Euclidean distance between pairs of cells, we were able to determine the distributions of distances for each pair of cell types. Of note, for every interaction measured (CD4^+^ with CD8^+^ T cells, TAMs with tumor, TAMs with CD4^+^ T cells, and TAMs with CD8^+^ T cells), the average distances between cells in tumors from PWH compared with those from PWOH, despite similar numbers of cells within each cell population (Supplemental Table 4), statistically significant differences in minimum distances to the tumor were observed for all 3 immune cell populations (Figure 6B), with the greatest effect size seen for CD68 cells (*r* = 0.51), followed by CD8 cells (*r* = 0.28). Statistically significant differences were also observed for minimum distances between immune cell populations, with the greatest effect size seen in the distances between CD8^+^ and CD4^+^ T cells (*r* = 0.31). In actual distances, the cell populations that displayed the largest distance were between TAMs and all other cell types (CD4^+^ and CD8^+^ T cells and CK^+^ tumor cells), as well as the distance between CD4^+^ and CD8^+^ T cells. The latter was likely reflected by the lower CD4/CD8 ratio among HIV-associated tumors. In addition, we measured the distances between CK^+^ tumor cells and various clusters of CD8^+^ T cells, CD4^+^ T cells, or CD68^+^ TAMs (Supplemental Figure 3). Effect sizes were smaller following clustering of immune cell clusters, probably due to the decreased variance.

Of note, the distance between tumor cells and CD8^+^ cluster 6, a cluster that demonstrated high levels of GZRB and low levels of exhaustion markers and thus likely to represented a function T cell population (Figure 3D), was significantly greater in tumors from PWH compared with those from PWOH (*P* < 0.001, *r* = 0.14). With regard to the CD4^+^ T cell subsets, only CD4^+^ cluster 4 showed a significantly increased distance from tumor cells in PWH compared with PWOH (*P* = 0.05). There was no difference in the distance between any of the CD68^+^ TAM clusters and tumor cells in PWH versus PWOH.

The use of neighborhood enrichment analysis revealed important differences between the organization of immune cells and tumor cells within the TME of PWH versus PWOH (Figure 6C). In this analysis of CD4^+^ T cells, we noted, for example an enrichment of cluster 6 CD4^+^ T cells close to CK^+^ tumor cells within the tumors from PWOH, which was not noted in tumors from PWH. Conversely, clusters 3 and 5 were enriched in tumors from PWH but not in those from PWOH. The CD8^+^ T cell subset neighborhood enrichment analysis was notable for showing greater enrichment of multiple CD8^+^ T cell clusters with tumor cells in the PWOH group compared with the PWH group — most notably CD8^+^ T cell clusters 1, 2, and 5. The CD68^+^ subset neighborhood enrichment analysis was notable for greater enrichment of CD68^+^ cluster 2 with tumor cells in the HIV^–^ group. There is greater enrichment of CD68 clusters 3, 6, and 7 with tumor cells in the HIV^+^ group.

### T cell–mediated tumor killing is impaired in PWH compared with PWOH.

**Figure 7:**
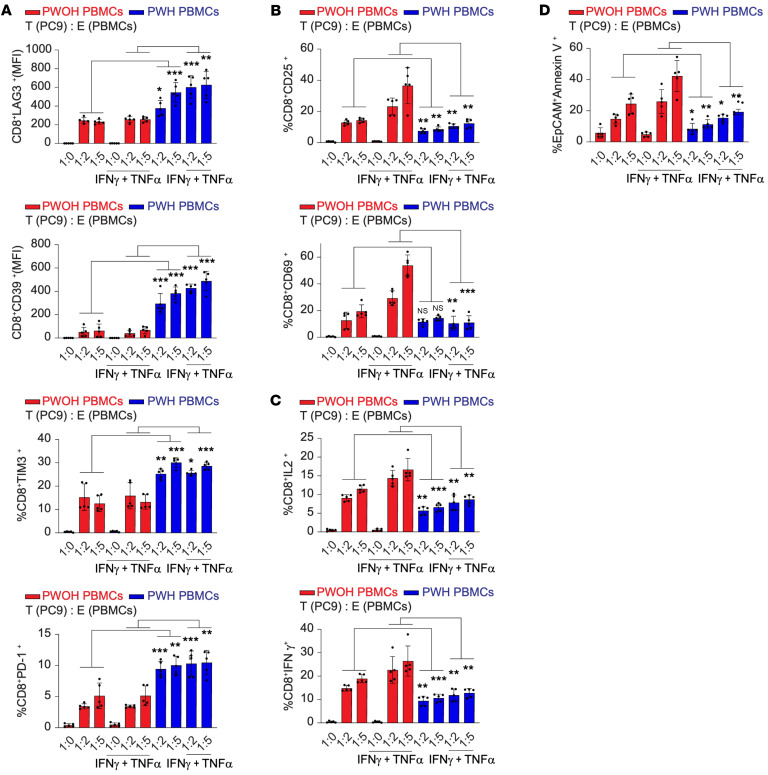
Tumor-killing assay with circulating cells from PWH and PWOH. PBMCs from PWOH (blue; *n* = 5) and PWH (red; *n* = 5) were incubated with PC9 tumor cells, an HLA-A2^+^ EGFR-mutant NSCLC tumor cell line, that were either in their native state or stimulated for 24 hours with IFN-γ and TNF-α. The T/E T cell ratio was 1:0, 1:2, or 1:5. The *y* axis represents the proportion of CD8^+^ T cells that expressed the exhaustion markers LAG3, TIM3, PD-1, or CD39 on CD8^+^ T cells (**A**); the proportion of CD8^+^ T cells expressing the activation markers CD25 and CD69 on CD8^+^ T cells (**B**); the proportion of CD8^+^ T cells that produced IL-2 or IFN-γ cytokines (**C**); and the proportion of EPCAM^+^ tumor cells expressing annexin V, a marker of cell death (**D**), upon exposure to PBMCs from PWH or PWOH. Data are presented as the mean ± SD. *P < 0.05, **P < 0.01, and ***P < 0.001, by 2-tailed unpaired Student’s *t* test with Holm-Bonferroni correction for multiple comparisons.

Tumor cells from the tumor cell line PC9 (HLA-A2^+^ EGFR mutant NSCLC) were either cocultured with PBMCs in their native state or after stimulation with IFN-γ and TNF-α to increase antigen presentation. Tumors were then exposed to PBMCs from donors (PWH or PWOH who expressed HLA-A1) at a 2:1 or 5:1 PBMC/tumor ratio. After 3 days of coincubation, we measured tumor killing by annexin staining of EPCAM^+^ tumor cells, and exhaustion was measured by LAG3, PD-1, TIM3, and CD39 expression on CD8^+^ T cells. Upon exposure to tumor cells, T cells from PWH showed significantly increased expression of exhaustion markers (LAG3, PD-1, TIM3, and CD39) but decreased expression of activation markers (CD25 and CD69) compared with T cells from PWOH (Figure 7, A and B). CD8^+^ T cells from PWH also expressed lower levels of IL-2 and IFNg upon co-culture (Figure 7C). This difference was noted when PBMCs were cocultured with tumor cells in their native state or after stimulation with IFN-γ and TNF-α. Strikingly, tumor cells in the presence of PBMCs from PWH had significantly lower expression of annexin V, a measure of cell death, compared with tumor cells cultured in the presence of PBMCs from PWOH (Figure 7D). When cultured with PBMCs from PWOH, we observed a dose-dependent increase in tumor cell killing (i.e., more annexin V staining with a 1:5 tumor/effector cell ratio). In PWH, an increase in the ratio of effector cells did not improve tumor killing, regardless of whether tumors were in their native state or stimulated with cytokines to increase antigen presentation. These findings suggest that cells from PWH, despite viral suppression, had an increased susceptibility to T cell exhaustion when encountering a neoantigen and also revealed deficiencies in tumor killing. Further confirmation of T cell dysfunction in the setting of HIV included decreased cytokine production (IL-2 and IFN-γ) in response to exposure to tumor cells. It is possible that some of the response to tumors was not antigen specific but may have been an alloresponse due to HLA mismatch. However, given that there was no matching to other HLA alleles in cells from either PWH or PWOH, the increased exhaustion markers and decreased tumor killing, activation, and cytokine production in response to an alloantigen in PWH also suggest a dysfunctional immune state. We observed similar results with a second tumor cell line, A549, which is an HLA-A2^+^ KRAS-mutant NSCLC cell line (Supplemental Figure 4).

All PWH in this analysis were on ART (median time on ART: 17 years) and virally suppressed (i.e., viral load [VL] undetectable and/or <20 copies/mcL). The median CD4^+^ T count was 673 cells/mcL. The median age of PWH was 66 years and 68 years for PWH. Among the PWH, 1 was White and 4 were Black. Among the PWOH, 3 were White and 2 were Black.

## Discussion

Among people living with HIV in the United States, NSCLC, a non-AIDS-defining cancer, is the leading cause of cancer-related death and occurs at a higher rate than in the general population (2, 3). Immune dysfunction in the setting of HIV infection is associated with chronic inflammation, both targeted to the virus as well as widespread bystander inflammation (22, 23). In addition, disruption of lymphatic tissue architecture, including lymph nodes, thymus, and bone marrow, further affect the ability to rectify years of immunologic damage even after viral suppression with ART (24–26). Global ongoing immune activation and immune dysfunction have been associated with increased all-cause mortality, and specifically with an increased cancer incidence (27–29). However, the effect of HIV-associated immunologic changes on the TME has not been studied and may explain the clinical finding of a worse cancer-associated prognosis for PWH. This may be particularly true for those malignancies, including NSCLC, in which we know that the immune response is an important indicator of prognosis. Using a well-matched cohort of NSCLC tumor tissue from PWH and PWOH, and IMC analysis with preserved tissue architecture, we found that PWH harbored a TME that was consistent with highly exhausted, dysfunctional CD4^+^ and CD8^+^ T cells. Furthermore, TAMs exhibited a program consistent with fostering a highly immunoregulatory environment. These findings demonstrate a clear differential effect of HIV infection on the immune microenvironment. Importantly, we have shown that T cells from PWH had a lower threshold to upregulate the immunoregulatory receptors LAG3, TIM3, PD-1, and CD39 and, strikingly, were less able to induce tumor killing of HLA-matched tumor cells, demonstrating that PWH, even after viral suppression, harbored both phenotypic and functional differences that could affect the response to neoantigens. Overall, both circulating immune cells and those within the tumor tissue of PWH showed clear evidence of fostering a regulatory immune environment, consistent with impaired antitumor immunity.

The TME is an important niche where tumor cells and surrounding immune and stromal cells provide bidirectional signaling that establishes either a tumor-promoting or tumor-regulatory environment. Although the number of TILs, particularly effector CD8^+^ T cells, in the TME has been associated with improved survival in several cancers including NSCLC (30), TIL numbers alone do not explain the differences between HIV-associated and non-HIV NSCLC, given the comparable levels of CD4^+^, CD8^+^, and TAM T cell infiltration seen in our study. In fact, there was a trend toward increased CD8^+^ T cells in HIV-associated NSCLC. However, a decreased CD4/CD8 ratio within the TME suggests that decreased help from CD4^+^ T cells for CD8^+^ effector cells may be a specific regulatory factor in HIV-associated tumors, as it is in the circulation. The CD4/CD8 ratio is inverted early after HIV infection, and its persistence after ART has been associated with increased cancer incidence and all-cause mortality, emphasizing this specific feature of HIV-associated immunologic perturbation as impactful in antitumor responses. In this analysis, we additionally found that immune cells (CD4, CD8, and CD68) had an increased distance to tumor cells in HIV-associated tumors, using Euclidean distance as well as neighborhood enrichment analysis in order to assess the spatial relationships between different cell types. Such differences may partially account for the differences in clinical outcomes.

In addition, we found that in PWH, CD8^+^ T cells within the TME had a dysfunctional phenotype, with increased expression of PD-1 and other inhibitory receptors (e.g., LAG3, TIM3) on T cells, rendering them less able to perform cytotoxic killing. The use of ICBs, such as those targeting the PD-1/PD-L1 pathway, has resulted in significant improvements in outcomes for some patients (31). However, the heterogeneity of expression of inhibitory receptors in the TME of patients has demonstrated that patients who harbor particularly “dysfunctional” or “exhausted” T cells are less likely to respond to ICBs. The recently described Ebo a cell subtype revealed an apoptosis-resistant, proliferative, but dysfunctional, T cell population with evidence of multiple inhibitory receptors including PD-1, LAG3, and TIM3 (12). The presence of Ebo cells has been associated with a poor response to ICB therapies. In this study, we found that HIV-associated tumors had striking increases in Ebo cells in both CD4^+^ and CD8^+^ T cell subsets, compared with non-HIV tumors. Accumulation of effector-like, apoptosis-resistant CD8^+^ T cells has been well described in the peripheral blood of HIV-infected individuals, secondary to a chronic hyperinflammatory state. We surmise that this global phenomenon observed in PWH is thus further accentuated in the TME, allowing tumors to grow with decreased immune pressure.

Parallel to changes in the adaptive immune responses (i.e., T cells), TAMs from HIV-associated tumors demonstrated some of the most striking differences compared with non-HIV tumors. CD68^+^ TAMs from PWH displayed multiple ligands from the B7 family, including PD-L1 (B7-H1), PD-L2 (B7-H2), B7-H3, and B7-H4, which bind to receptors on T cells that regulate the cytolytic and cytotoxic responses of the TAMs. The expression of PD-L2 was particularly elevated across TAMs from HIV-associated tumors and was, in fact, the primary marker identified using spectral graph theory that distinguishes HIV-associated tumors versus non-HIV tumors. This finding raises the possibility of exploring PD-L2 as a potential target in HIV-associated tumors. In addition, TAMs from HIV-associated tumors also overexpressed VISTA, another immune checkpoint marker, as well as IDO1, an enzyme associated with metabolic changes occurring in immunoregulation. Although PD-L1, a current target of several ICBs used clinically, is also increased among HIV-associated TAMs, the highly increased expression across other members of the B7 family, as well as VISTA and IDO1, suggests that anti-PDL1 strategies may be less effective in PWH due to multiple overlapping pathways by which regulatory signaling to T cells is augmented, further contributing to poor antitumor immunity. We additionally noted that β-2-microglobulin was increased in TAMs from PWH, suggesting possible enhanced antigen presentation in the HIV-associated samples, and acknowledge the need for further phenotyping with inclusion of additional markers of immune activation. These combined phenotypes of immune activation and immune regulation have been described in HIV and are a critical and unique feature of this chronic viral infection, in which years of antigenic viral exposure results in the activation of immune cells. However, given the chronicity and broad tissue scope of antigenic exposure, the mounting of an effective immune responses is, ultimately, impaired. Our TME and peripheral blood data further illustrate these dual features of immune dysregulation in the setting of HIV.

The role of IMC as a methodology that incorporates simultaneous measurement of multiple markers also allows for dynamic characterization of the TME. Using both cell segmentation and segmentation-independent approaches, we have concordance in the findings that HIV-associated NSCLC had evidence of a more immunoregulatory landscape. AI-based approaches were also able to differentiate between HIV-associated and -uninfected tumors with greater than 80% accuracy in this small dataset, with PD-L2 (on TAMS) and CD25 (on T cells), respectively, being the most discriminating features of an HIV-associated TME. These analyses suggest that targeting immune cells in the TME, an approach that is increasingly being embraced in drug development, may require specific strategies for PWH, in whom the differences in immune cells are distinct. Further refinement of markers and larger datasets may result in even more robust differentiation between these 2 groups.

Spatial organization of cells within the TME additionally revealed distinct differences in the distances between immune cells and tumor cells within tumors from PWH compared with those from PWOH.

Our finding that T cells from PWH, even after durable viral suppression on ART, had a higher likelihood of upregulating immune-regulatory receptors and a decreased capacity for tumor killing, provides further evidence that circulating immune cells in PWH have a decreased ability to become activated in the presence of tumor neoantigens and decreased cytotoxicity, likely due to decreased thresholds to upregulate markers of exhaustion. Although this methodologic approach may demonstrate some immune responses that are not HLA restricted due to a lack of matching for all MHC class I/II alleles, the increased expression of exhaustion markers in this setting, regardless, emphasizes that this functional defect is likely to affect the ability of PWH to respond to neoantigens in vivo, including in the tumor environment of malignancies like NSCLC, which we know are dependent on an effective immune response to control tumor growth. This impaired ability of PBMCs to become activated or demonstrate functionality is also consistent with prior reports (32).

Using data on a cohort of PWH and PWOH with NSCLC from a single institution, matched for cancer stage, histology, age, tobacco use, and year of diagnosis, we performed a comprehensive evaluation of the TME among HIV-NSCLC tumors using multiple imaging modalities and bioinformatics analytic platforms, this study systematically assessed the diversity of the TME in NSCLCs among PWH and uninfected well-matched controls. In order to differentiate specific features of HIV infection and their effects on outcome (e.g., HIV control, different ART regimens, duration of HIV infection at the time of cancer diagnosis), a much larger dataset will be necessary and should be pursued. Another limitation of our findings is the inability to match for ancestry and sex, an important caveat that will require a future, larger multi-institutional cohort for analysis. Our findings demonstrate that the TME of HIV-associated NSCLC was fundamentally different from that of non-HIV NSCLC and that circulating T cells from PWH, even after effective HIV control, had a decreased ability to kill tumor cells. These findings are critically important for a patient population at higher risk for NSCLC, who present at a younger age and have a worse prognosis. Importantly, our study identifies unique targets for this patient population that require further evaluation in larger-cohort studies. These differences also highlight the need for PWH to be included in clinical trials for ICB therapies in order to evaluate responses in this unique patient population. The similarity of the TME features among those with HIV-associated NSCLC and those who respond poorly to ICBs in the general population suggests that HIV-NSCLC may further illuminate the features of NSCLC that reflect a nonresponse to current immune-based approaches.

## Methods

### Sex as a biological variable.

There was no exclusion of participants on the basis of sex: both female and male participants were included in this study.

### Patients, samples, and tissue microarray construction.

Formalin-fixed, paraffin-embedded (FFPE) NSCLC tissue from 18 PWH who underwent surgery/biopsy at Yale New Haven Hospital between January 2001 and January 2016 were obtained from the archives of the Pathology Department at Yale University. NSCLC biopsies from an additional 19 PWOH infection served as controls and were matched on the basis of major clinicopathologic factors including cancer stage, histologic subtype, year of biopsy, age, smoking status, and sex. Patients designated as not having HIV either had no history of HIV infection and/or documented negative HIV testing or absence of HIV testing in the medical record. A tissue microarray (TMA) was constructed from 0.6 mm tissue cores of each FFPE sample with 2-fold redundancy as previously reported by our group (9–12, 14). Lymph node, tonsil, and placental tissue samples were included in the TMA to serve as positive controls for TIL markers and PD-L1 signal.

### IMC.

Preparation of the TMA and antibody cocktail for IMC analysis was performed using a modified IMC Staining Protocol for FFPE Sections from Fluidigm. The TMA slide was first incubated at 60°C for 30 minutes in a slide oven, incubated at room temperature for 20 minutes in fresh xylene under a fume hood for dewaxing twice, and then hydrated with ethanol solutions of descending concentrations for 1 minute each. The TMA was then washed with tap water for 5 minutes on a shaker plate and incubated at 96°C in the antigen retrieval solution (1 mM EDTA at a pH of 9) for 20 minutes, followed by cooling to 70°C for 10 minutes. The TMA was incubated in a blocking solution containing 0.3% BSA in TBS-T (Tween-20 0.05%) for 30 minutes at room temperature.

An antibody cocktail containing metal-conjugated antibodies (DNA-191/193Ir, vimentin-143Nd, TBET–145Nd, CD47-146Nd, pan-CK–148Nd, CD45Ro-149Sm, PDL1-150Nd, GAPDH 151Eu, B7H3-152Sm, LAG3–153Eu, TIM3–154Sm, FOXP3-155Gd, CD4-156Gd, B7-H4-158Gd, CD68-159Tb, PD1-160Gd, CD20-161Dy, CD8-162Dy, CD25-163Dy, VISTA-165Ho, KI67–168Er, B2M-169Tm, CD3-170Er, IDO1-171Yb, PDL2-172Yb, granzyme B–173Yb, histone 3–176Yb) diluted in 0.3% BSA in TBS-T was prepared. The TMA slide was incubated in the antibody cocktail diluted in BSA 0.3% TBS-T solution overnight at 4°C; washed twice with 0.05% TBS-T at room temperature on a shaker plate for 5 minutes each time; incubated for 30 minutes in a solution containing intercalator-Ir191/Ir193 (diluted 1:2,000 in TBS-T) for 60 minutes at room temperature; washed for 5 minutes in TBS-T twice, followed by a wash for 5 minutes in MQ grade ddH2O twice; and finally left to air-dry at room temperature for 30 minutes, protected from humidity and sunlight. Data for IMC were acquired with a Hyperion instrument (DVs, Fluidigm Sciences). All antibody targets, conjugated metals, and final working concentrations of solutions prepared are listed in Supplemental Table 1.

For IMC analysis, extraction of the raw IMC marker score and development of tissue images were done using MCD Viewer, version 1.0.560.0 (Fluidigm). Unsupervised cell segmentation and mask generation were completed using CellProfiler, version 3.1.8. Briefly, cell nuclei were identified as primary objects within CellProfiler using positive DNA expression on TIFF images created in MCD Viewer for each tissue sample. Cell segmentation was automated and based on distance approximation using a standard radius of 3 pixels from all previously identified primary objects (identified as nuclei by DNA intercalators and histone 3 staining). If 2 cells overlapped in an area, the cell boundaries were approximated using the median distance between their nuclei. The optimal distance threshold of 3 pixels was set after comparing cell segmentation results using a range of radii with manually identified cell boundaries on TIFF images. A mask image was generated, which outlined the boundaries of each unique cell identified via segmentation. Segmented cell masks from CellProfiler were extracted and imported into histoCAT (Histology Topography Cytometry Analysis Toolbox), version 1.75, along with the corresponding TIFF images for each marker of interest in each tissue sample. PhenoGraph was used for unsupervised clustering through the MATLAB-based tool CYT, and single-cell data were extracted for analysis (33). PhenoGraph partitions a matrix of single-cell measurements into subpopulations on the basis of their phenotypic similarity (expression of markers) and builds this graph by finding the nearest neighbors for each cell (using Euclidean distance), resulting in N sets of *k* neighborhoods. It then builds a weighted graph, such that the weight between nodes scales with the number of neighbors they share, as described in ref. 33. FlowJo, version 10.0.7, was used for gating of cell populations, identifying CK^–^CD68^–^CD20^–^CD3^+^CD4^+^CD8^–^, CK^–^CD68^–^CD20^–^CD3^+^CD4^–^CD8^+^, CK^–^CD68^+^CD20^–^CD3^–^, and CK^+^CD68^–^CD20^–^CD3^–^ cell populations from IMC single-cell data.

### Spectral graph theory.

For every tumor within the TMA, 14 patches of size 35 μm² were selected and centered at the highest CK expression level. For each patch, the “stack” of expression levels across all antibodies was examined, and using graph analysis, a feature vector was constructed. Then, the feature vectors of all patches from all cases were assembled into a representation matrix, in which the rows corresponded to the number of markers and the columns to the number of patches. This matrix was then subjected to a nonlinear dimensionality reduction using diffusion maps. Subsequently, the matrix in the reduced dimension was used to predict the HIV status. For the prediction, we used an RBF SVM classifier and leave-one-case-out cross-validation analysis. In this process, the classifier was trained with all the patches, except those from 1 case, and then tested on the excluded patches.

### In vitro tumor-killing assay.

A549 and PC9 cells were cultured in DMEM and RPMI 1640 respectively, supplemented with 10% FBS and antibiotics (10,000 U/mL penicillin, 10 μg/mL streptomycin), followed by incubation at 37°C and 5% CO_2_. Exponentially grown cells were used in this study. Cell lines were authenticated every 3–6 months according to laboratory protocols. A549 and PC9 cells were left unstimulated or stimulated with IFN-γ and TNF-α (20 ng/mL each) for 24 hours before being washed and cocultured in the presence of PBMCS (at a 2:1 or 5:1 PBMC/tumor ratio) for 72 hours. PBMCs were obtained from HLA-02^+^ donors (PWH, *n* = 3 and PWOH *n* = 3). After incubation, cocultured cells were stained to analyze T cell markers, CD8 (BD, catalog 566852; BD, catalog 555368), CD25 (BD, catalog 560987), CD69 (BD, catalog 555530), LAG3 (BioLegend, catalog 369306), and PD-1 (BD, catalog 560795), as well as the tumor cell markers EPCAM (BioLegend, catalog 369306) and annexin V (BioLegend, catalog 640906) as per the manufacturer’s instruction. The cells were analyzed with a LSR II flow cytometer (BD Biosciences). Subsequent analysis was performed using FCS Express software version 7 (Devovo Software).

### Cluster proportions and fold change analysis.

SciPy, version 1.13.1 (34), was used to calculate cluster proportions, determine cluster fold changes (FCs), and perform Fisher’s exact tests to assess proportional changes. Heatmaps illustrating the FC in cell cluster proportions in biopsies from PWH compared with biopsies from PWOH were created using Scimap, version 2.1.2 (35).

### Spatial distance calculation and analysis.

The *x*, *y* spatial coordinates of each cell were extracted, and the minimum Euclidean distances between cell types were calculated using SciPy. The distributions of these distances were visualized with violin plots using Seaborn, version 0.13.2 (36). Wilcoxon testing was performed using SciPy for comparison of distance distributions between PWH and PWOH. Effect size (*r*) was calculated as the *Z* statistic divided by the square root of the total number of pairwise distance measurements between cells (*r* = Z/√N). Kernel density estimates of the probability density of distances were visualized using Seaborn.

### Neighborhood enrichment analysis.

Neighborhood enrichment analysis assesses the spatial relationships between different cell types and determines whether certain cell types are more likely to be neighbors than would be expected by chance. The analysis entails construction of a spatial connectivity graph based on spatial coordinates of cells, calculation of enrichment scores for each pair of cell types, and *Z* scaling. Distance metrics are used to define local neighborhoods. The connectivity matrix consists of weighted edges between the cells (i.e., nodes) — spatial weights depend on the distance between the cells. The enrichment score is calculated for each pair of cell types and reflects how often 2 different cell types are neighbors compared with a random distributions of cells. Positive enrichment scores indicate that cell types co-occur in local neighborhoods more often than would be expected by chance. Negative enrichment scores indicate that cell types co-occur in local neighborhoods less often than would be expected by chance.

Subsets consisting of tumor cells and CD4, CD8, or CD68 clusters were created and further stratified by HIV status. The *x*, *y* spatial coordinates of each cell were extracted. Construction of spatial neighbors graph and subsequent neighborhood enrichment analysis were performed separately for PWH and PWOH groups using Squidpy, version 1.5.0 (37).

### Statistics.

The IMC signal from non-HIV and HIV-associated patients were compared using a Dunn’s multiple-comparison test if statistical significance was found in a nonparametric Kruskal-Wallis test, an unpaired 2-tailed Student’s *t* test, a Mann-Whitney *U* test, or Welch’s correction and a χ^2^ test for continuous and categorical variables, respectively. For Figure 3, B and E, and Figure 4B, statistical analyses were conducted using a linear mixed-effects model to account for between-individuals variability. Individual effects were modeled as random effects to appropriately capture the hierarchical structure of the data. All analyses were performed using the lmerTest package (version 3.1-3) in R (version 4.2.2), and statistical significance was determined at a threshold of 0.05. For Supplemental Figure 2, the statistical significance of differences in the proportions of immune cell subsets was evaluated using the nonparametric Wilcoxon rank-sum test (Wilcox.test function in R). To assess changes in proportions of clusters between PWOH and PWH, the Fisher’s exact test was performed using SciPy (version 1.13.1). The difference in expression of annexin on tumor cells or inhibitory receptors on T cells in the functional assays was assessed using an unpaired 2-tailed Student’s *t* test, with adjustment for multiple comparisons. For Figure 7 and Supplemental Figure 4, differences between groups were determined by 2-tailed, unpaired Student’s *t* test, with a Holm-Bonferroni correction for multiple comparisons.

### Study approval.

Approval of the Yale University’s Human Investigations Committee (HIC) was granted for all studies that involved humans. Written informed consent was obtained from all participants for human studies that were deemed nonexempt. The collection and analysis of samples used in this study were approved by the Yale University HIC (protocol no. 1608018220).

### Data availability.

All data will be made available upon request. Values for all data points in the graphs are provided in the Supporting Data Values file.

## Author contributions

SSD conducted experiments, acquired and analyzed data, and edited the manuscript. SS conducted experiments, acquired and analyzed data, and wrote the manuscript. RY designed research studies, conducted experiments, and acquired and analyzed data. KR conducted experiments. JG analyzed data and edited the manuscript. LO analyzed data and edited the manuscript. YWL, SM, and RT analyzed data. IK and YK analyzed data and edited the manuscript. HZ acquired and analyzed data and edited the manuscript. KS designed research studies, analyzed data, and edited manuscript. BE designed research studies, analyzed data, and wrote the manuscript. The first 3 authors contributed equally to the manuscript and are listed alphabetically.

## Supplementary Material

Supplemental data

Supporting data values

## Version 1 06/03/2025

In-Press Preview

## Version 2 07/15/2025

Electronic publication
