# Supplementary material for: The tumor microenvironment of non–small cell lung cancer impairs immune cell function in people with HIV
Source: J Clin Invest. 2025 Jun 3;135(14):e177310. doi: 10.1172/JCI177310 (PMC12259253; doi:10.1172/JCI177310)
Supplement: Supplemental data [file jci-135-177310-s230.pdf]

Supplemental Table 1: Antibodies for Immune Mass Cytometry

Channel	Metal	Target	Clone	Vendor
143	Nd	Vimentin	RV202	Fluidigm
145	Nd	Tbet	D6N8B	Fluidigm
146	Nd	CD47	B6H12.2	Novus/Fluidigm
148	Nd	pan-CK	AE1/AE3	Fluidigm
149	Sm	CD45Ro	UCHL1	Fluidigm
150	Nd	PDL1	E1L3N	Fluidigm
151	Eu	GAPDH	D16H11	CST/Fluidigm
152	Sm	B7H3	D9M2L	CST/Fluidigm
153	Eu	Lag3	D2G4O	Fluidigm
154	Sm	Tim3	D5D5R	Fluidigm
155	Gd	FoxP3	236A/E7	Fluidigm
156	Gd	CD4	EPR6855	Fluidigm
158	Gd	B7H4	D1M8I	CST/Fluidigm
159	Tb	CD68	KP1	Fluidigm
160	Gd	PD-1	EH33	CST/Fluidigm
161	Dy	CD20	H1	Fluidigm
162	Dy	CD8	C8/144B	Fluidigm
163	Dy	CD25	GLLZDMY	ThermoFisher/Fluidigm
165	Ho	Vista	D1L2G	CST/Fluidigm
168	Er	Ki67	B56	Fluidigm
169	Tm	B2M	D8P1H	Fluidigm
170	Er	CD3	POLY-RB	Fluidigm
171	Yb	IDO-1	D5J4E	CST/Fluidigm
172	Yb	PDL-2	D7U8C	Fluidigm
173	Yb	Granzyme B	496B	ThermoFisher/Fluidigm
176	Yb	Histone3	D1H2	Fluidigm
Costaining				
192/193	Ir	DNA	HI30	

*Custom conjugated at Fluidigm

Supplemental Table 2: Patient Demographics

Patient Characteristics	HIV-NSCLC N=18		Uninfected N=19		
	N	%	N	%	P-value
Median age	54.1		57.2		0.22
Gender					0.06
Male	14	78%	9	47%	
Female	4	22%	10	53%	
Race and Ethnicity					0.02
White, Non-Hispanic	6	33%	15	79%	
Black, Non-Hispanic	9	50%	2	11%	
Hispanic	3	17%	1	5%	
Other/Unknown	0	0%	1	5%	
Smoking Status					0.49
Non-Smoker	0	0%	2	11%	
Smoking History	18	100%	17	89%	
Year of Diagnosis					0.70
1999-2004	4	22%	6	32%	
2005-2010	8	44%	6	32%	
2011-2016	6	33%	7	37%	
Histology					0.77
Adenocarcinoma	8	44%	10	53%	
Squamous Cell	8	44%	8	42%	
NSCLC (unspecified)	2	11%	1	5%	
Stage at diagnosis					0.96
Stage I	6	33%	6	32%	
Stage II	1	6%	2	11%	
Stage III	4	22%	4	21%	
Stage IV	7	39%	7	37%	
Years of HIV Diagnosis					
Median	17	-			
Range	2-36	-			
AIDS Diagnosis	8	44%			
HIV VL (copies/mL)					
<400	10	56%			
>400	8	44%			
CD4 count (cells/µL)					
Median	440	-			
Range	26-872	-			

Supplemental Table 3: Individual Demographics of Patients

Study ID	Age	Sex	HIV status	Viral Load**	Race and Ethnicity	Stage	Smoking	Histology	Treatment*
1	61	Male			White, Non-Hispanic	4	Current	Squamous cell carcinoma	R, C, P
2	61	Male			White, Non-Hispanic	4	Prior	Adenocarcinoma	S
3	59	Male			Hispanic	4	Current	Squamous cell carcinoma	S, R, C
4	49	Male			White, Non-Hispanic	4	Current	NSCLC, NOS	S, R, C
5	53	Female			White, Non-Hispanic	4	Current	Adenocarcinoma	S, R, C
6	61	Male			White, Non-Hispanic	4	Prior	Adenocarcinoma	C
7	68	Male			Black, Non-Hispanic	4	Never	Adenocarcinoma	S, I
8	32	Female			Other/Unknown	1A	Unknown	NSCLC, NOS	S
9	55	Female			White, Non-Hispanic	1A	Unknown	Adenocarcinoma	S
10	62	Female			White, Non-Hispanic	1A	Current	Squamous cell carcinoma	S
11	67	Female			White, Non-Hispanic	1A	Current	Squamous cell carcinoma	S
12	52	Female			White, Non-Hispanic	1A	Prior	Adenocarcinoma	S
13	51	Male			White, Non-Hispanic	1B	Never	Adenocarcinoma	S, C
14	62	Female			White, Non-Hispanic	2A	Current	Adenocarcinoma	S, C
15	57	Female			White, Non-Hispanic	2A	Prior	Adenocarcinoma	S, C
16	53	Female			Black, Non-Hispanic	3A	Current	Adenocarcinoma	S, C
17	65	Female			White, Non-Hispanic	3A	Current	Squamous cell carcinoma	S, C
18	54	Male			White, Non-Hispanic	3A	Prior	Squamous cell carcinoma	S, C
19	65	Male			White, Non-Hispanic	3B	Current	Squamous cell carcinoma	S, C
20	64	Male	PWH	ND	White, Non-Hispanic	1A	Past	Adenocarcinoma	S
21	47	Male	PWH	<50	Black, Non-Hispanic	1B	Current	NSCLC, NOS	S
22	62	Female	PWH	276	Black, Non-Hispanic	1A	Current	Squamous cell carcinoma	
23	54	Female	PWH	<20	Black, Non-Hispanic	1A	Prior	Adenocarcinoma	S
24	50	Male	PWH	42,224	White, Non-Hispanic	4	Current	Adenocarcinoma	R, C
25	60	Male	PWH	349	Hispanic	3A	Current	Squamous cell carcinoma	R, C
26	52	Male	PWH	189,000	Black, Non-Hispanic	4	Prior	NSCLC, NOS	R, P
27	43	Male	PWH	31000	Black, Non-Hispanic	4	Current	NSCLC, NOS	
28	46	Female	PWH	5120	Hispanic	1A	Current	NSCLC, NOS	S
29	48	Male	PWH	25190	Black, Non-Hispanic	3B	Current	NSCLC, NOS	R, C
30	58	Male	PWH	<48	White, Non-Hispanic	3A	Current	NSCLC, NOS	C
31	60	Male	PWH	734	Black, Non-Hispanic	4	Current	NSCLC, NOS	S, R, C
32	52	Female	PWH	37,000	White, Non-Hispanic	4	Current	NSCLC, NOS	P
33	48	Male	PWH	<50	White, Non-Hispanic	1B	Current	NSCLC, NOS	R, C
34	55	Male	PWH	<20	Black, Non-Hispanic	3B	Current	Squamous cell carcinoma	C
35	64	Male	PWH	107	Hispanic	2A	Current	Adenocarinoma	C
36	62	Male	PWH	ND	White, Non-Hispanic	4	Prior	Adenocarcinoma	R, C, P
37	49	Male	PWH	5628	Black, Non-Hispanic	4	Current	NSCLC, NOS	

*S=Surgery; C=Chemotherapy; R=Radiotherapy; P=Palliative

**ND=non-detectable

Supplemental Table 4: T cell Number within TME

	P value	Mean (HIV)	Mean (non-HIV)	Difference	SE of difference	t ratio	df	q value
CK+	0.170988	1236	1564	328.8	237.3	1.386	60	0.703621
CD8+	0.083642	170.2	72	-98.16	55.54	1.767	47	0.697019
CD4+	0.253303	57.74	94.84	37.11	32.1	1.156	49	0.703621
CD68+	0.371457	115.8	82.43	-33.32	36.97	0.9014	53	0.773868

Supplementary Figure 1: Survival Data among Patients with HIV-associated NSCLC

A

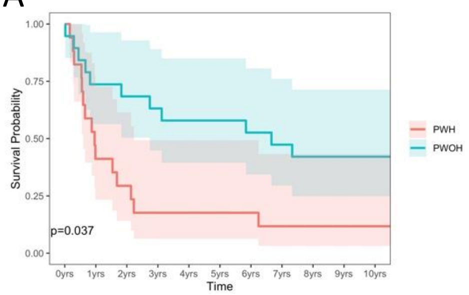

B

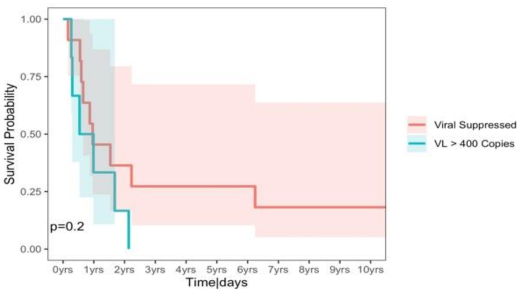

Supplemental Figure 1: (A) 10-year survival among PWH (n=18) and PWOH (n=19) from diagnosis of NSCLC. p=0.037 (B) 10-year survival among PWH with HIV load > 400 copies/mcl (n=8) and PWH with suppressed viral load (HIV load < 400 copies/mcl; n=10) at time of cancer diagnosis. P=0.20

Supplementary Figure 2: Proportions of Immune Cell Subsets between PWH and PWOH

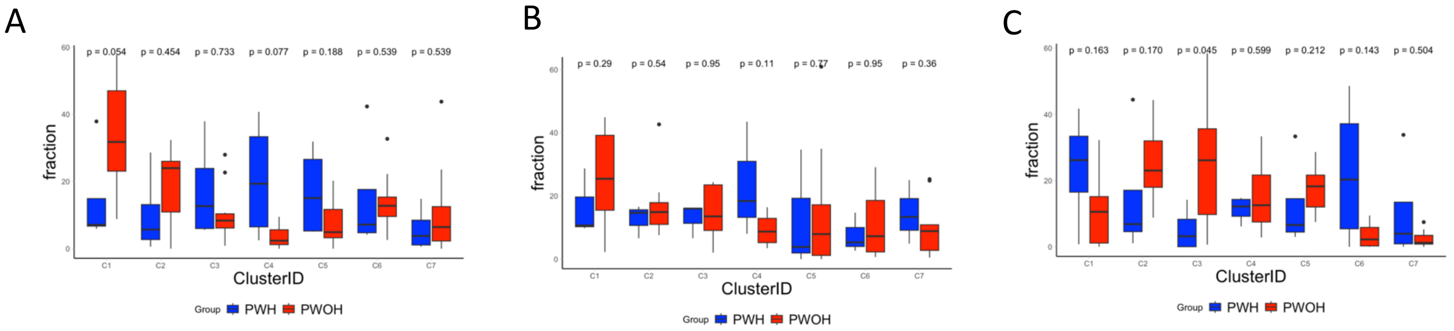

Supplemental Figure 2: (A) 10-year survival among PWH and PWOH from diagnosis of NSCLC. p=0.037 (B) 10-year survival of PWH with evidence of high proportion (>10% of total CD8+ T cells), of an Effector Burned out, “Ebo” phenotype compared to those with a low proportion (<10%).

Supplementary Figure 3: Distance between Tumor Cells and Immune Cell Subsets

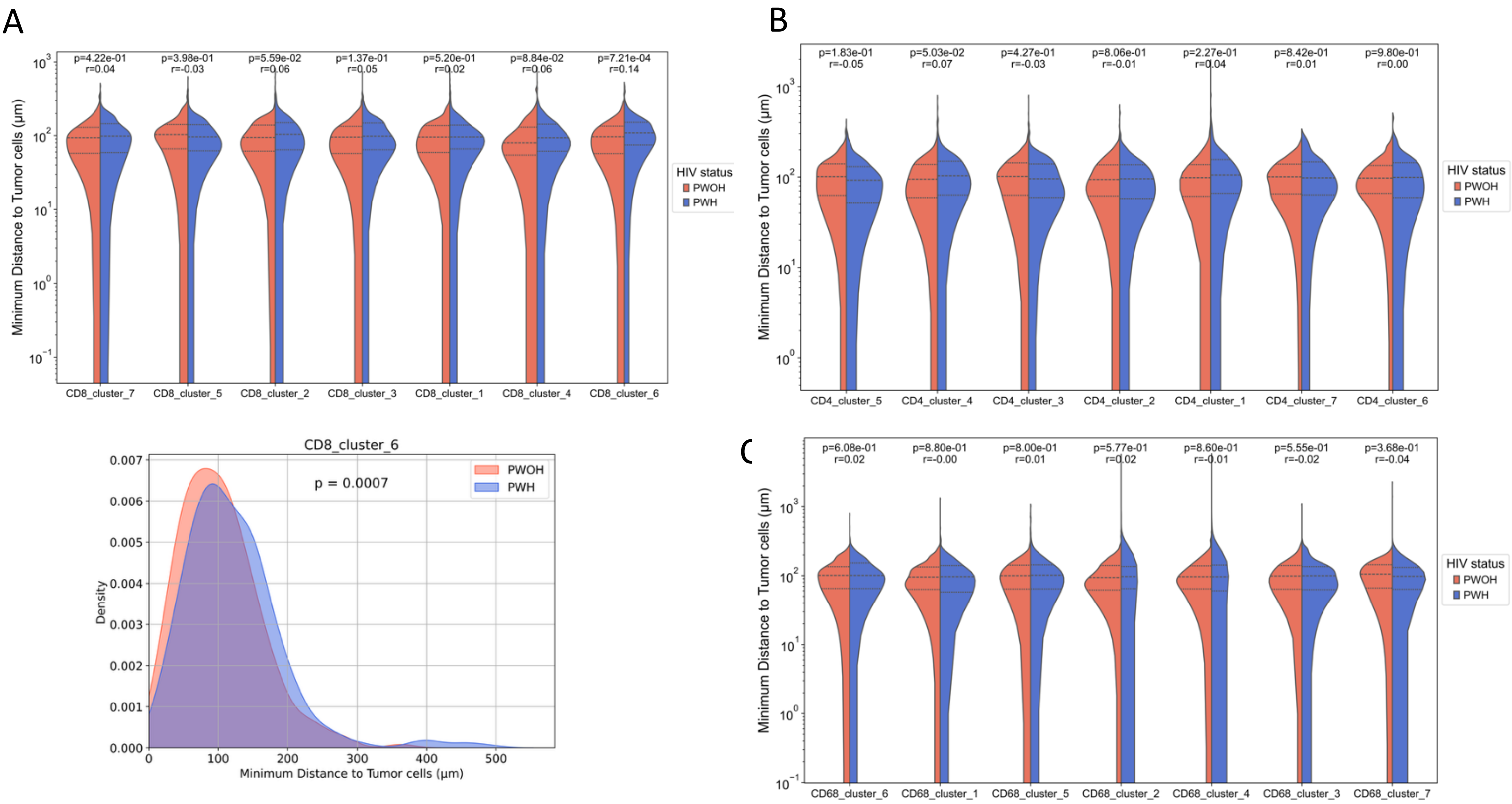

Supplemental Figure 3: (A) Split violin plots of minimum Euclidean distances (log transformed) between CD8 subsets and CK+ tumor cells. (B) Kernel density estimate plot of minimum distances of CD8 cluster 6 cells to tumor cells. Split violin plotsof minimum Euclidean distances (log transformed) between (C) CD4 subsets and CK+ tumor cells and (D)) CD68 subsets and CK+ tumor cells . Wilcoxon test used for comparison of distributions between PWOH and PWH.

Supplementary Figure 4: Tumor Killing Assay with Circulating Cells from PWH and PWOH (A549)

T-cell dysfunction marker

Apoptotic cell death marker

T-cell activation marker

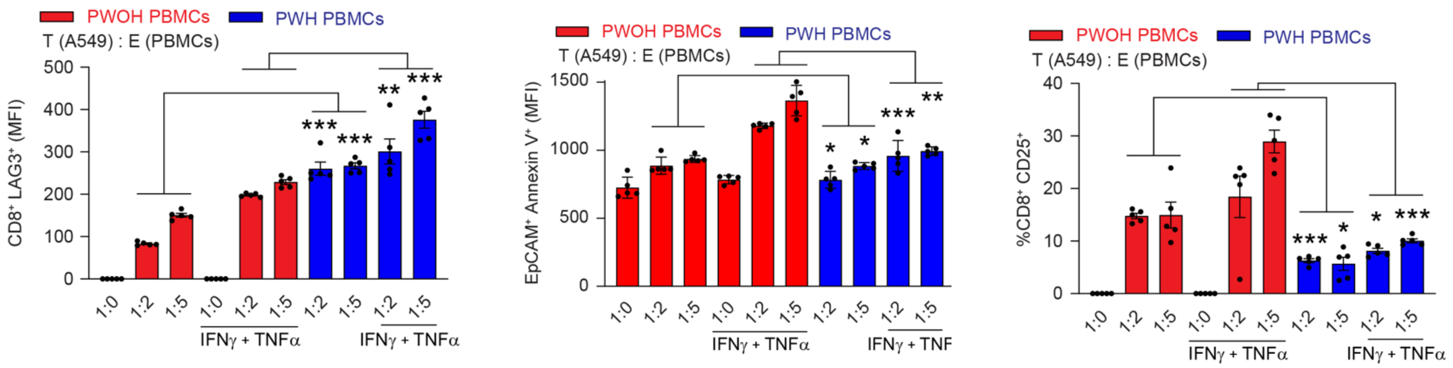

Supplemental Figure 4: PBMCs from PWH (n=5) and PWOH (n=5) were incubated with tumor cells from tumor cell line A549 and were either in their native state or stimulated for 24 hours with IFNg and TNFa. Tumor:effector T cell ration was 1:0, 1:2, or 1:5. Y-axis represents the proportion of CD8+ T cells that expressed T cell dysfunction marker Lag3, proportion of EpCAM+ tumor cells that expressed cell death marker Annexin V, or CD8+ T cells that expression T cell activation marker CD25. Data presented as the mean +/- s.d.. * represents $p<0.05$; ** represents $p<0.01$; *** represents $p<0.001$ as determined by two-tailed unpaired Student’s t test, with a Holm-Bonferroni correction for multiple comparisons.
